# Cytomegalovirus in Plasma of Acute Coronary Syndrome Patients

**Published:** 2016

**Authors:** E. A. Nikitskaya, J.C. Grivel, E. V. Maryukhnich, A. M. Lebedeva, O. I. Ivanova, P. P. Savvinova, A. V. Shpektor, L. B. Margolis, E. Yu. Vasilieva

**Affiliations:** Laboratory of Atherothrombosis, Cardiology Department, Evdokimov Moscow State University of Medicine and Dentistry, Delegatskaya str. 20/1, 127473, Moscow, Russia;; Section on Intercellular Interactions, Eunice Kennedy Shriver National Institute of Child Health and Human Development, National Institutes of Health, Bethesda, MD, USA; Current affiliation: Sidra Medical and Research Center, P.O. Box 26999, Doha, Qatar

**Keywords:** coronary artery disease, acute coronary syndrome, human herpes viruses, cytomegalovirus, polymerase chain reaction

## Abstract

The relationship between acute coronary syndrome (ACS) and local and systemic inflammation, including accumulation of macrophages in atherosclerotic plaques and upregulation of blood cytokines (e.g., C-reactive protein (CRP)), has been known for more than 100 years. The atherosclerosis-associated inflammatory response has been traditionally considered as an immune system reaction to low-density lipoproteins. At the same time, some data have indicated a potential involvement of cytomegalovirus (CMV) in the activation and progression of atherosclerosis-associated inflammation, leading to ACS. However, these data have been tangential and mainly concerned the relationship between a coronary artery disease (CAD) prognosis and the anti-CMV antibody titer. We assumed that ACS might be associated with CMV reactivation and virus release into the bloodstream. The study’s aim was to test this assumption through a comparison of the plasma CMV DNA level in patients with various CAD forms and in healthy subjects. To our knowledge, no similar research has been undertaken yet. A total of 150 subjects (97 CAD patients and 53 healthy subjects) were examined. Real- time polymerase chain reaction (RT-PCR) was used to determine the number of plasma CMV DNA copies. We demonstrated that the number of plasma CMV genome copies in ACS patients was significantly higher than that in healthy subjects (*p *= 0.01). The CMV genome copy number was correlated with the plasma CRP level (*p* = 0.002). These findings indicate a potential relationship between CMV activation and atherosclerosis exacerbation that, in turn, leads to the development of unstable angina and acute myocardial infarction. Monitoring of the CMV plasma level in CAD patients may be helpful in the development of new therapeutic approaches to coronary atherosclerosis treatment.

## INTRODUCTION

Atherosclerotic coronary artery lesions often lead to the development of a coronary artery disease (CAD) that manifests itself as angina or painless myocardial ischemia. This disease can last for years as stable coronary artery disease (SCAD) forms, with occasional exacerbations presenting themselves clinically as unstable angina or acute myocardial infarction (AMI). These clinical manifestations are grouped under the name of acute coronary syndrome (ACS). The morphological substrate of this exacerbation is supposed to be acute inflammation followed by atherosclerotic plaque rupture and thrombosis formation [1-3]. Despite the fact that the role of inflammation in the development and progression of atherosclerosis has been under discussion for the second century running since the time of Virchow [4], the causes of this inflammation are not completely clear. The very fact of an inflammation is confirmed by the presence of macrophages and lymphocytes in the plaques, an elevated level of inflammatory cytokines in atherosclerosis patients, etc. [5-10]. According to the most accepted theory, the primary trigger of an inflammatory reaction in the vascular wall is the subendothelial accumulation of oxidized low-density lipoproteins [11-13]. At the same time, there are data indicating that atherosclerotic plaques contain various bacteria and viruses [14-19] that can also induce an inflammatory response. Herpesviruses, in particular the cytomegalovirus (CMV), are of huge interest. Many epidemiological studies have revealed a relationship among the incidence of coronary atherosclerosis, the incidence of acute myocardial infarction, and the blood level of anti-CMV antibodies [20, 21]. However, this is insufficient to assess the viral infection activity during atherosclerosis exacerbation. An exception is a study by S. Gredmark *et al*. [22], demonstrating that CMV RNA in the monocytes of ACS patients occurs more often than in those of healthy donors and patients with chronic forms of CAD, which may indicate activation of the virus during ACS. At the same time, no direct analysis of the plasma CMV level in patients with atherosclerotic coronary artery disease has been previously performed. The presence of the virus in plasma may indicate its activation [23-25]. In this work, we present a comparative study of CMV in plasma of patients with various forms of CAD and healthy volunteers.

## MATERIALS AND METHODS

**Characterization of groups of patients and healthy volunteers**

The study involved 150 participants, including 97 CAD patients and 53 healthy volunteers. Seventy-one patients were admitted to the Cardiac Critical Care Department of the Davydovskiy Municipal Clinical Hospital with a diagnosis of acute coronary syndrome. Of these, 47 patients were diagnosed with AMI with or without ST-segment elevation in accordance with the universal definition of the European Society of Cardiology [26]; unstable angina was diagnosed in 24 cases. Twenty-six patients were admitted electively. CAD was diagnosed based on the clinical picture and positive stress test results, which was later confirmed by coronary angiography [27]. In all patients, the clinical prognosis was evaluated; there were no cases of death, hemodynamically significant bleeding, stroke, or stent thrombosis. At admission, two ACS patients were diagnosed with cardiogenic shock; two patients had acute heart failure; 12 patients had acute left ventricular aneurysm; seven patients with a severe coronary artery disease had repeated angina attacks.

An examination of healthy volunteers included a survey, blood chemistry, ultrasound of the heart and carotid arteries, and a stress test. According to the examination data, no subjects with signs of atherosclerosis were identified in the control group.

**Table 1:** Clinical characteristics of CAD patients and healthy volunteers

Indicator	ACS patients	SCAD patients	Healthy volunteers	p
Number of patients	71	26	53	
Mean age	64.4 ± 9.7	66.3 ± 10.6	61.3 ± 12.3	0.116
Males	63.4%	65.4%	50.9%	0.298
Smoking	28.2%	11.5%	20.8%	0.205
Hyperlipidemia	35.2%	15.4%	34.0%	0.135
Obesity	45.1%	23.1%	15.1%	0.001^*^
Hypertension	90.1%	92.3%	47.2%	0.000^*^
Diabetes mellitus	31.0%	19.2%	1.9%	0.0002^*^

The clinical characteristics of all three groups of patients are presented.

^*^Differences are statistically significant at p < 0.05.

Patient groups did not differ in age or gender, but they differed in the presence of risk factors, such as obesity, arterial hypertension, and diabetes (*Table 1*).

All participants provided a written informed consent to participate in this study. The study was approved by the local ethics committee of the Evdokimov Moscow State University of Medicine and Dentistry.

**Isolation of viral DNA from plasma**

In all patients, a 5-mL blood sample was collected into a test tube with sodium citrate within 24 h after admission. Blood samples were centrifuged at 2,500 rpm for 10 min, after which the plasma was collected, frozen in sterile test tubes, and stored at –80°C until further use.

The samples were thawed, and DNA was isolated from the plasma using QIAamp DNA Blood mini kit columns (Qiagen, Germany) according to a standard protocol. Elution was performed using 60 μL of a special buffer from the same kit. Before conducting the real-time polymerase chain reaction (RT-PCR), DNA samples were stored at –20°C.

**Quantitative RT-PCR**

CMV was detected by RT-PCR (CFX 96 C1000 Touch Thermal Cycler, Bio-Rad, USA) using highly sensitive primers and a 5’-3’-hydrolyzable probe to the CMV tegument protein pp65 gene (*Table 2*). Amplification was evaluated from the standard curve using standard dilution series (Bioresearch Technologies, USA) and ToughMix PCR mixtures (Quanta, USA, Cat # 95147-250).

**Table 2:** CMV primers and probes

Probe/Primer	Nucleotide sequence	5’-modification	3’-modification
Probe	tacctggagtccttctgcgagga	CAL Fluor Red 610^*^	BHQ-2^**^
Forward primer	aaccaagatgcaggtgatagg		
Reverse primer	agcgtgacgtgcataaaga		

^*^CAL Fluor Red 610 is a fluorescent label on the probe.

^**^BHQ-2 is a fluorescence quencher.

RT-PCR was performed according to the standard three-step protocol: step 1 – denaturation at 95°C for 5 min, step 2 – 95°C for 30 s, and step 3 – 60°C for 60 s.

Next, the fluorescence signal was detected.

The second and third steps were again repeated for 45 cycles. Fluorescence detectable up to the 37th cycle was considered specific. The results were presented as the CMV DNA copy number in 1 μL of the patient blood plasma.

**Measurement of the high-sensitivity C-reactive protein (hs-CRP)**

At admission, all patients underwent an analysis of hs- CRP, whose level is correlated with the risk of cardiovascular events [28]. The protein plasma level was determined on an automatic analyzer (Siemens Dimention Xpand Plus, Germany) using a C-Reactive Protein Flex Reagent kit (Siemens # DF37, Germany).

**Statistical data processing**

The statistical analysis was performed using the Statistica 9.0 software. All obtained data had no signs of a normal distribution based on the Shapiro-Wilk test and, therefore, were represented as median and interquartile ranges. Because of the non-parametric distribution, the Mann-Whitney test was used for comparison between two groups. Non-parametric statistics with the Kruskal-Wallis test and multiple comparison rank test were used to compare more than two groups. The Spearman correlation coefficient was also used. Differences between groups were considered statistically significant at the level of *p * < 0.05.

## RESULTS AND DISCUSSION

Small CMV DNA concentrations (over 100 copies in 1 μL of blood plasma) were quite frequently found both in patients and in healthy volunteers. The rate of virus detection in the three groups differed statistically significantly and was highest in ACS patients (*Table 3*).

**Table 3:** The CMV occurrence rate in different groups

	Healthy volunteers	ACS patients	SCAD patients	p
Number of virus-positive patients	46.15% (18/39)	77.08% (37/48)	55.56% (10/18)	0.013

**Fig. 1:**
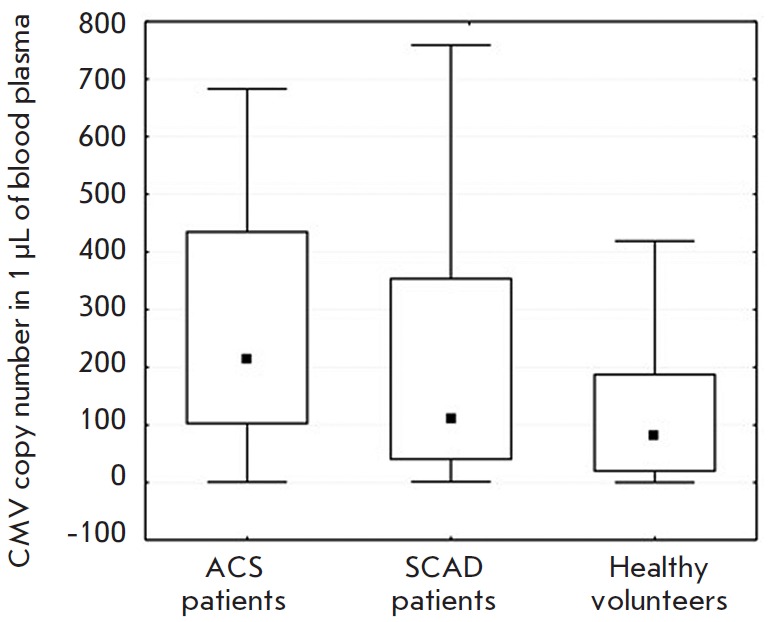
Comparison of the CMV DNA copy number in the blood plasma of patients in the study groups. The median and 25th–75th percentiles of the CMV DNA copy number in the three groups are presented. Statistically significant differences were found between the ACS group and the healthy volunteer group (p = 0.012).

**Fig. 2:**
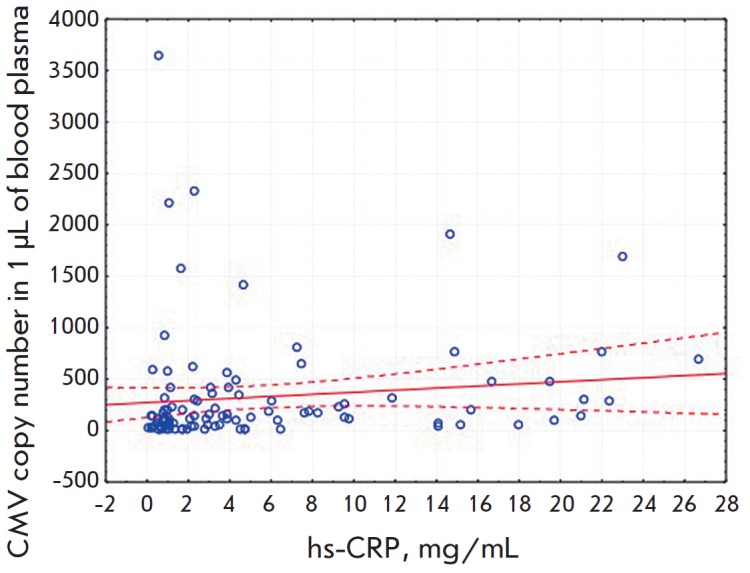
A correlation between the CMV genome copy number and the hs-CRP level is shown. Results of an individual analysis of the CMV copy number and hs-CRP level are presented. A correlation between the indicators with 95% confidence intervals is demonstrated. Two samples exceeding the mean cohort values by almost 10 times were excluded from the analysis. The correlation coefficient R was 0.25 (p = 0.011) before excluding the samples and 0.30 (p = 0.002) after exclusion.

Comparison of the number of CMV DNA copies in three groups revealed significant differences between ACS patients and healthy volunteers (213.15 [101.21–436.67] versus 82.10 [18.58–188.67], respectively,* p *= 0.012). However, no statistically significant differences between the group of chronic CAD patients and the group of healthy volunteers were found. The results are shown in *Fig. 1*. In addition, a statistically significant (*p *= 0.002) positive correlation between the number of CMV copies and the hs-CRP level was found in this cohort (*Fig. 2*).

Therefore, we had demonstrated that the occurrence and number of CMV copies in the blood plasma of patients with acute CAD forms were significantly higher than those in healthy controls. No differences between the chronic CAD group and the control group were found.

These findings demonstrate that a small amount of the virus is quite often present in the plasma of healthy individuals (*Table 3*). This is consistent with epidemiological study data on a CMV-seropositive adult population in various countries [24, 29-30]. Our data indicate that the number of CMV DNA copies can substantially increase in pathology: in the case of ACS, the number was more than 2 times higher than that in healthy volunteers. Our findings are generally consistent with epidemiological data on the correlation between the presence of CMV and atherosclerosis progression, with allowance for the anti-CMV antibody titer [31]. For example, one of the epidemiological studies had revealed a correlation between cardiovascular disease mortality and the anti-CMV antibody titer level [17]. An ARIC study also showed that cardiovascular disease mortality was proportional to an increase in the carotid artery intima-media thickness [32]. However, results of seroepidemiological studies are contradictory. For example, a prospective, controlled study by P.M. Ridker* et al*. revealed no relationship between the presence of anti-CMV antibodies and the risk of atherothrombotic events. In this case, the antibody titer height was not evaluated separately [33].

Previously, the herpesvirus DNA was identified in plaques and blood monocytes by PCR [34]. Melnick *et al*. [35] demonstrated for the first time that CMV DNA was present in the artery walls of atherosclerosis patients. The viral DNA concentration was higher in the arterial wall of patients who underwent reconstructive vascular surgery (coronary artery bypass grafting) compared to patients with early atherosclerosis [36]. Later, CMV was found in the atherosclerotic plaques [37]. We also studied samples obtained from patients who had died of acute myocardial infarction or its complications, but we did not find significant differences in the number of CMV DNA copies in the atherosclerotic plaques and coronary arteries without macroscopic signs of atherosclerosis [38].

The inconsistency of these data may be associated with the fact that both CMV seropositivity and the presence of CMV DNA in tissues and blood cells are not sufficient to conclude on virus replication. In the present work, the number of CMV DNA copies was determined in the plasma of patients with various CAD forms. The presence of the virus in plasma indicates productive infection [23, 24, 29-30]. Another indicator of productive infection may be the presence of CMV RNA, which was detected in peripheral blood mononuclear cells [22]. The amount of CMV RNA in blood monocytes of ACS patients was significantly higher than that in stable angina patients and healthy subjects (*p * < 0.001). In this case, the occurrence of CMV RNA in monocytes was relatively small and amounted to 2% in healthy volunteers, 10% in SCAD patients, and 15% in ACS patients [22]. In general, these data are consistent with the results of our work. However, the occurrence rate of the virus in our groups was higher, possibly due to the fact that blood monocytes are not the only body cells secreting CMV into the plasma.

The morphological basis of ACS is an atherosclerotic plaque rupture, probably due to inflammation in the plaque. A number of studies using histochemical techniques have demonstrated that the plaques contain activated lymphocytes and macrophages [11, 12, 39-42]. Previously, we used an original technique for isolation of cells from the plaque, preserving cell surface antigens, and their evaluation by flow cytometry [43]. This enabled us to quantitatively evaluate the number of activated lymphocytes (CD8^+^CD25^+^ and CD8^+^HLA-DR^+^) in the plaques, which happened to be significantly higher than that in the blood. In other studies, along with those by our group, a number of bacteria and viruses, including CMV, were found in blood vessels using RT-PCR [38]. This may be the cause of chronic activation of the immune system in vessels, stimulating the development of atherosclerosis [44]. The role of either oxidized lipoproteins or microorganisms in this activation remains unclear. It may not be excluded that detection of viruses in blood vessels is not related to atherosclerosis itself. They may be present in the vascular wall without playing any pathogenetic role in the development of this pathology. The data obtained in this study disprove this assumption: an elevated CMV DNA level in the plasma of ACS patients indicated enhanced virus replication upon atherosclerosis exacerbation. It is not clear whether the CMV activation plays the major role in the atherosclerosis progression, or other microorganisms may also be involved in this process. Also, the relationship between two factors, CMV reproduction and hyperlipidemia, has not been determined yet. A combination of both mechanisms is possible: CMV reproduction in the plaque may be accompanied by more active lipoprotein accumulation by macrophages. Lipoproteins subjected to oxidization, in turn, may enhance inflammatory reactions in the vascular wall. To answer these questions, further research is needed. The promising area seems to be further analysis of the CMV plasma level in patients with various forms of coronary atherosclerosis and comparison of the virus level with changes in the disease clinical picture.

## CONCLUSION

Thus, we have demonstrated the fact of CMV activation in ACS patients. The number of CMV DNA copies in the plasma is correlated with the level of hs-CRP, a systemic inflammation marker. CMV activation is probably one of the mechanisms triggering the inflammatory process in the atherosclerotic plaque, which leads to disruption of the plaque integrity and subsequent thrombus formation. Further investigation of the mechanisms of CMV effects on atherosclerosis progression may be helpful in developing new approaches to the treatment of CAD.

## Abbreviations

HHV: human herpes virus
hs-CRP: high-sensitivity C-reactive protein
CAD: coronary artery disease
AMI: acute myocardial infarction
SCAD: stable coronary artery disease
ACS: acute coronary syndrome
RT-PCR: real time polymerase chain reaction
CMV: cytomegalovirus
